# Supplementary material for: Identification and functional analysis of non-coding regulatory small RNA FenSr3 in Bacillus amyloliquefaciens LPB-18
Source: PeerJ. 2023 May 15;11:e15236. doi: 10.7717/peerj.15236 (PMC10194069; doi:10.7717/peerj.15236)
Supplement: Supplemental Information 4 [file peerj-11-15236-s004.zip › KO/CK-vs-T1_map/map04978.html]

KEGG PATHWAY: Mineral absorption - Reference pathway


|  |  |
| --- | --- |
| **Mineral absorption - Reference pathway** |  |

[
Pathway menu
| Organism menu
| Pathway entry
| Show description
| User data mapping
]

Minerals are one of the five fundamental groups of nutrients needed to sustain life. Of the minerals, calcium plays innumerable roles in our bodies, serving as a main component of bone as well as an intracellular messenger in muscle contraction/relaxation, neural networks, the immune system, and endocrine/exocrine cells. Iron, copper, and other metals are required for redox reactions (as cofactors) and for oxygen transport and binding (in hemoglobin and myoglobin). Many enzymes require specific metal atoms to complete their catalytic functions. Animal tissues need moderate quantities of some elements (Ca, P, K, Na, Mg, S, and Cl) and trace amounts of others (Mn, Fe, I, Co, Cr, Cu, Zn, and Se). The minerals are absorbed by either passive or active transport systems through the intestinal mucosa, often using specialized transport proteins, such as ferritin for Fe3+ and vitamin D-induced protein for calcium.

|  |  |  |
| --- | --- | --- |
| Reference pathway | 184% 150% 122% 100% 82% 67% 55% | 图片下载 |
